# Pharmaceutical Innovations: The Grand Challenges Ahead

**DOI:** 10.3389/fmedt.2019.00003

**Published:** 2020-01-09

**Authors:** Rita I. Aroeira, Miguel A. R. B. Castanho

**Affiliations:** Instituto de Medicina Molecular, Faculdade de Medicina, Universidade de Lisboa, Lisbon, Portugal

**Keywords:** innovation, therapy, diagnosis, device, medicine, drugs, diseases

Lifestyles are evolving rapidly due to swiftly evolving technologies rapid exchange of information across the globe, and facilitated mobility of people across long distances. In addition, climate changes accelerate the geographical dynamics of disease. The result is that both communicable and non-communicable diseases pose challenges never faced before and the perception of the way pharmaceutical sciences are dealing with such changes is under unprecedented scrutiny. The discredit in science-based solutions has a tremendous societal impact and is detrimental to evidence-based pharmacology at large. Pharmaceutical innovation that target the needs of healthy living and meet the expectation of society are urgently needed and are a worthy effect of both industrial and academic researchers. A reflection on the grand challenges ahead in thus timely and appropriate.

## Expanding the Borders

### Brain and CNS

The brain and the rest of the Central Nervous System (CNS) are the last big frontier of biomedical knowledge. Compared to other organs and systems, such as the heart/circulatory system, the brain/CNS is largely unknown in its fundamentals. Structure-function relationships and cell-cell communication has far more unknown in the brain than in other human organs. Recently, research institutes devoted to study the brain have been created, informal networks of researchers as the European Brain Council were developed, and many millions in research grants and biotech programs are being invested, but we are barely starting to pave the way (1).

### Aging and Re/De-generation

While the power to expand longevity leaves no doubt, the way to avoid or treat neurodegeneration remains elusive. Despite the huge effort to understand and manage Alzheimer's disease and other dementias, for instance, the limited knowledge on the biochemistry and physiology of the brain undermines establishing standardized procedures and, consequently, potentiate conflicting results (2). Living longer and healthier will remain a top priority for decades.

### Man/Device Interface

Material science and nanotechnology endowed health sciences with new tools and opportunities that hold promises for innovation in Pharmaceutical Sciences. Cell-machine sensor communication and mutual responsiveness is of utmost importance in cardiology and neurology, for instance. Brain circuitry modulation to treat brain diseases and spinal cord injury recovery are among the most important and impactful topics in modern medicine (3, 4).

## Reliable and Predictive Models

Take standard procedures for drug development today as an example. Their efficacy is debatable as well as their capacity to respond rapidly to the urgent need of new medicines against emerging of quickly evolving diseases. One statement is consensual: they are extremely expensive and generate a lot of waste—waste of time, waste of money, waste of data, and waste of research consumables. What alternatives can be devised to reduce waste and robustly deliver safe and efficient medicines? The answer remains to be known.

### *In vitro, ex-vivo*, or *in vivo* Veritas?

Ethics and cost-efficacy investments call for *in vitro* systems able to circumvent *in vivo* tests. Nevertheless, in virtually all domains of pharmacology *in vivo* testing cannot be totally replaced by *in vitro* approaches. While *in vitro* testing in being successfully used to pre-screen drug candidates thus reducing the burden of *in vivo* testing, efficacy and, above all, safety evaluation of new chemical entities still demand heavy *in vivo* testing (5). Reverting this limitation remains a goal but there is not yet convincing evidence it is within reach.

### Clinical Trials

With the prevailing idea that the only good model for human physiology and pharmacology are … humans (!), there is raising claim that regulation on clinical trials, mainly early stage clinical trials (“first in human” tests), need flexibility to encourage innovation in chemical and biological therapies. Despite the challenges and ethical considerations in designing a protocol for clinical trials, research-driven clinical trials, while in close scrutiny, should be facilitated so that more reliably data on human response to new therapies can be gathered and processed (6).

## The New Landscape of Disease

Climate changes, so does the ecology of pathogens. Human gastronomy changes, so does the evolution of metabolic diseases. Human attitudes changes, so does the reemergence of formerly nearly eradicated diseases. Human lifestyle changes, so does life expectancy. Altogether, the world map of endemic malignancies is changing fast as subtropical pathogens (e.g., Dengue virus) expand north, high caloric diets make obesity a public health threat in increasing areas of the globe, compliance to anti-vaccine ideologies enabled reappearance of once controlled diseases such as measles (7), for instance, and cardiovascular diseases menace reverting life expectancy soon.

### The Revenge of Microbials

Together with vaccines, antimicrobials were probably the most important achievement in Medicine, ever. Yet, the advent of antimicrobial resistance by bacteria, viruses, and other microorganisms, as well as the advent of boycotts to vaccination policies, created a very serious health threat (8). New drugs not prone to resistance and new strategies for their use are urgently needed.

### Cardiovascular and Metabolic Malignancies

Obesity, diabetes, and metabolic syndrome constitute a deadly triad in wealthy regions of the globe. Coping with prophylaxis and treatment is badly needed but extremely demanding. This is fertile ground for pharmaceutical innovation.

## Scaling Up and Getting Smarter

### Smart Molecules, Smart Materials, and Smart Drug Delivery

Developments in medicinal chemistry, instrumental analysis, and biological engineering enabled a plethora of new chemical agents having properties that can be manipulated (e.g., self-assembly, temperature and pH-responsiveness, solubility in lipids,…) and have increased complexity (e.g., antibody-drug conjugates, peptide mimetics…). These new capacities, together with the use of nanotechnology, have translated in new self-delivered drugs, and high precision targeted drug delivery systems, thus advancing the old paradigm of the “magic bullet” (2, 9).

### From Small Molecules to Biologicals and Cell Therapies

Concomitantly to developments in the “magic bullets,” biological drugs (e.g., antibodies) came into play and cells hold a promise as future large-scale therapies. Pharmaceutical innovation opportunities reside on the side of biology as much as they reside on chemistry and nanotechnology (10, 11).

## “Undrugging” the Environment: Pharma Goes Green

Drug and drug metabolites release in the environment due to uncontrolled waste disposal and excretion by humans and animals (mainly farming animals) pose a serious environmental challenge. New solutions are needed both in waste reduction and management, and safe products of pharma industry (12). Innovation is badly needed to bring pharma into circular economy and eco-friendship.

## Conclusions

Innovation in pharmaceutical sciences is needed to meet current challenges imposed by societal and environmental demands. Although innovation is largely determined by the interplay of pharmaceutical sciences with other sciences such as biochemistry, biology, physiology, and electronic engineering, its interdisciplinarity is fertile ground for innovative approaches. While the nature and geography of diseases evolve rapidly, new responses have to the generated at equal pace.

Innovative therapeutic solutions are being developed to cope with a new landscape of diseases, using more reliable and predictive models of disease. Marking these therapeutics environment friendly and taking then beyond current borders of knowledge is a mission to be fulfilled, should the researchers in academia and industry not lose focus and dare to think and act outside the canonical boxes of consecrated standards.

## Author Contributions

MC conceived the paper structure, searched for information, and wrote the paper. RA searched for information and wrote the paper.

### Conflict of Interest

The authors declare that the research was conducted in the absence of any commercial or financial relationships that could be construed as a potential conflict of interest.
